# Enantioselective Analytical- and Preparative-Scale Separation of Hexabromocyclododecane Stereoisomers Using Packed Column Supercritical Fluid Chromatography

**DOI:** 10.3390/molecules21111509

**Published:** 2016-11-10

**Authors:** Nicole Riddell, Lauren Gayle Mullin, Bert van Bavel, Ingrid Ericson Jogsten, Alan McAlees, Allison Brazeau, Scott Synnott, Alan Lough, Robert McCrindle, Brock Chittim

**Affiliations:** 1 Wellington Laboratories Inc., 345 Southgate Drive, Guelph, ON N1G 3M5, Canada; alan@well-labs.com (A.M.); allison@well-labs.com (A.B.); scott@well-labs.com (S.S.); bob@well-labs.com or rmccrind@uoguelph.ca (R.M.); brockc@well-labs.com (B.C.); 2 Man-Technology-Environment (MTM) Research Center, Örebro University, 70182 Örebro, Sweden; Lauren.Mullin@oru.se (L.G.M.); Bert.VanBavel@oru.se (B.v.B.); Ingrid.Ericson@oru.se (I.E.J.); 3 Waters Corporation, 34 Maple Street, Milford, MA 01757, USA; 4 Norwegian Institute for Water Research, NO-349 Oslo, Norway; 5 Department of Chemistry, University of Toronto, Toronto, ON M5S 3H6, Canada; alough@chem.utoronto.ca; 6 Chemistry Department, University of Guelph, Guelph, ON N1G 2W1, Canada

**Keywords:** hexabromocyclododecane, enantiomeric separation, supercritical fluid chromatography

## Abstract

Hexabromocyclododecane (HBCDD) is an additive brominated flame retardant which has been listed in Annex A of the Stockholm Convention for elimination of production and use. It has been reported to persist in the environment and has the potential for enantiomer-specific degradation, accumulation, or both, making enantioselective analyses increasingly important. The six main stereoisomers of technical HBCDD (i.e., the (+) and (−) enantiomers of α-, β-, and γ-HBCDD) were separated and isolated for the first time using enantioselective packed column supercritical fluid chromatography (pSFC) separation methods on a preparative scale. Characterization was completed using published chiral liquid chromatography (LC) methods and elution profiles, as well as X-ray crystallography, and the isolated fractions were definitively identified. Additionally, the resolution of the enantiomers, along with two minor components of the technical product (δ- and ε-HBCDD), was investigated on an analytical scale using both LC and pSFC separation techniques, and changes in elution order were highlighted. Baseline separation of all HBCDD enantiomers was achieved by pSFC on an analytical scale using a cellulose-based column. The described method emphasizes the potential associated with pSFC as a green method of isolating and analyzing environmental contaminants of concern.

## 1. Introduction

The additive brominated flame retardant 1,2,5,6,9,10-hexabromocyclododecane (HBCDD) is utilized at percent levels in extruded and high-impact polystyrene foams and to a lesser extent in electrical equipment housings [1]. Although 16 stereoisomers are possible through bromination of cyclododeca-1,5,9-triene, the technical mixture is comprised mainly of three pairs of enantiomers (75%–89% γ-HBCDD, 10%–13% α-HBCDD, and 1%–12% β-HBCDD) with two additional meso forms having been reported to be present at very low levels (δ- and ε-HBCDD) [2]. In spite of having similar chemical structures, the isomers of HBCDD exhibit varying physical properties with water solubilities of 48.8, 14.7, and 2.1 μg/L for the α, β, and γ isomers, respectively [3]. Since HBCDD is not chemically bound to the products into which it is incorporated, movement into environmental matrices is possible; however, the variation of physicochemical properties among the stereoisomers leads to differential biological uptake and degradation rates resulting in environmental isomeric ratios that differ from those of the technical material. Indeed, biomagnification factors of greater than 1 have been reported for the α- and γ-HBCDD stereoisomers, and an increase in the proportion of α isomer has been observed with an increasing trophic level in the food web [2]. Reported environmental concentrations of the three main HBCDD diastereomers vary considerably depending on the matrix analyzed; however, in general, the relative isomeric proportions in abiotic matrices is usually similar to that of the commercial mixture with the γ isomer dominating, while biotic samples tend to be dominated by the α isomer [4,5]. It has been suggested that HBCDD diastereomer profiles in abiotic samples can be affected by thermal isomeric interconversion during product manufacture [6] and photolytically mediated processes [7], whereas profiles in biotic samples are largely affected by bioisomerization, isomer selective uptake, differential metabolism, and exposure [8]. Enantiomer specific analysis of HBCDD has generated significant interest since enzymes involved in the degradation of this environmental contaminant may be enantioselective as with other chiral POPs [9].

Both the developmental toxicity of HBCDD diastereomers in zebrafish [10] and the cytotoxicity on Hep G2 cells [11] follow the order γ-HBCDD > β-HBCDD > α-HBCDD; however, in addition to this trend, the cytotoxicity study published by Zhang et al. also reported significantly lower cell viability and higher LDH release for all three (+)-enantiomers [11]. Several enantiomer-specific studies have been published and non-racemic HBCDD distributions have been reported in bird [8,12,13] and fish [14] samples. Related studies on enantiomer-specific accumulation [15,16,17,18,19], isomerization [20,21,22,23], and degradation [24] of HBCDD diastereomers have also been conducted with many researchers relying on enantiomeric fraction (EF) calculations rather than characterized HBCDD enantiomer reference standards since such standards are not commercially available. Unfortunately, there are analytical challenges associated with isomer specific HBCDD analysis using liquid chromatography coupled with electrospray ionization mass spectrometry (LC-ESI/MS) such as differences between enantiomer ionization efficiencies (actual or artificial) [25], column bleed [26], and matrix effects [27] that can affect the accuracy of EF calculations if mass-labeled internal standards are not employed. It has also been noted that, although the enantiomers of each individual HBCDD stereoisomer (e.g., (+)- and (−)-α-HBCDD) can be effectively separated using published chiral LC methods, the simultaneous separation of all enantiomers (e.g., (+)-β-HBCDD from (+)-γ-HBCDD) is more difficult and can require two-dimensional techniques to achieve baseline separation [28]. The effective separation of HBCDD diastereomers using packed column supercritical fluid chromatography coupled with mass spectrometry (pSFC-MS) has recently been demonstrated [29]. The objective of this study was to investigate the enantiomeric separation and isolation of HBCDD stereoisomers using a similar separation technique and to characterize the isolated enantiomers using X-ray crystallography and LC retention time in comparison to published findings. Preparative scale separations of compounds such as the HBCDD isomers which have limited solubility in traditionally utilized solvents often result in large collection volumes for the isolation of small quantities of purified material. The advantages of using pSFC over preparative scale LC include reduced solvent usage and shorter equilibration times. Additionally, the superior solvating power of supercritical fluids [30,31] allows for potentially higher column loadings that can translate into fewer injections.

## 2. Results and Discussion

Multiple reports exist in the literature specifying the LC elution order of the HBCDD enantiomers on a Nucleodex β-PM stationary phase (permethylated β-cyclodextrin bonded to silica) as being (−)-α-HBCDD, (−)-β-HBCDD, (+)-α-HBCDD, (+)-β-HBCDD, (+)-γ-HBCDD, followed by (−)-γ-HBCDD [32,33]. Thorough descriptions of the absolute configurations of the (+)- and (−)-α, β-, and γ-HBCDD stereoisomers obtained through preparative chiral phase-LC have been published by both Koeppen et al. and Heeb et al. However, since the elution order of the diastereomers changes from α-HBCDD, β-HBCDD, and γ-HBCDD by LC-MS to α-HBCDD, γ-HBCDD, and β-HBCDD by pSFC-MS, it was prudent to repeat the characterization of the isolated pSFC fractions in order to definitively identify the elution order of the enantiomers under the stated pSFC-MS conditions since analytical standards were not available.

### 2.1. Preparative Separation of HBCDD Enantiomers

Prior to preparative separation, preliminary method development was performed on an analytical scale to determine optimal chiral stationary phases (CSPs) and cosolvents for effective separation of the target HBCDD enantiomers. Chiral column screening included evaluation of both amylose- and cellulose-based stationary phases (Waters Acquity UPC^2^ Trefoil AMY1 [amylose tris-(3,5-dimethylphenylcarbamate)], CEL1 [cellulose tris-(3,5-dimtheylphenylcarbamate)], and CEL2 [cellulose tris-(3-chloro-4-methylphenylcarbamate)] columns) using common pSFC solvents (methanol, acetonitrile, 2-propanol, and ethanol). The amylose stationary phase demonstrated ineffective separation of all HBCDD enantiomers using the stated cosolvents, but the cellulose-based columns gave promising results, so further method optimizations focused solely on the CEL1 and CEL2 columns. Baseline separation of all HBCDD enantiomers was accomplished using the analytical CEL2 column and a 2-propanol modified carbon dioxide mobile phase; however, when the method was transferred to a preparative scale, retention of the β-HBCDD enantiomers resulted in long run times and high percentages of cosolvent to effect elution. For this reason, the β-HBCDD enantiomers were separated and collected on the preparative scale using a shorter isocratic run on the CEL1 stationary phase that employed an acetonitrile modified carbon dioxide mobile phase. Optimized preparative scale methods resulted in baseline separation of the α-, β-, and γ-HBCDD enantiomers investigated, with enantiomeric purities of 100% being accomplished for all isolated compounds.

### 2.2. Characterization of Isolated HBCDD Enantiomers

**Figure 1:**
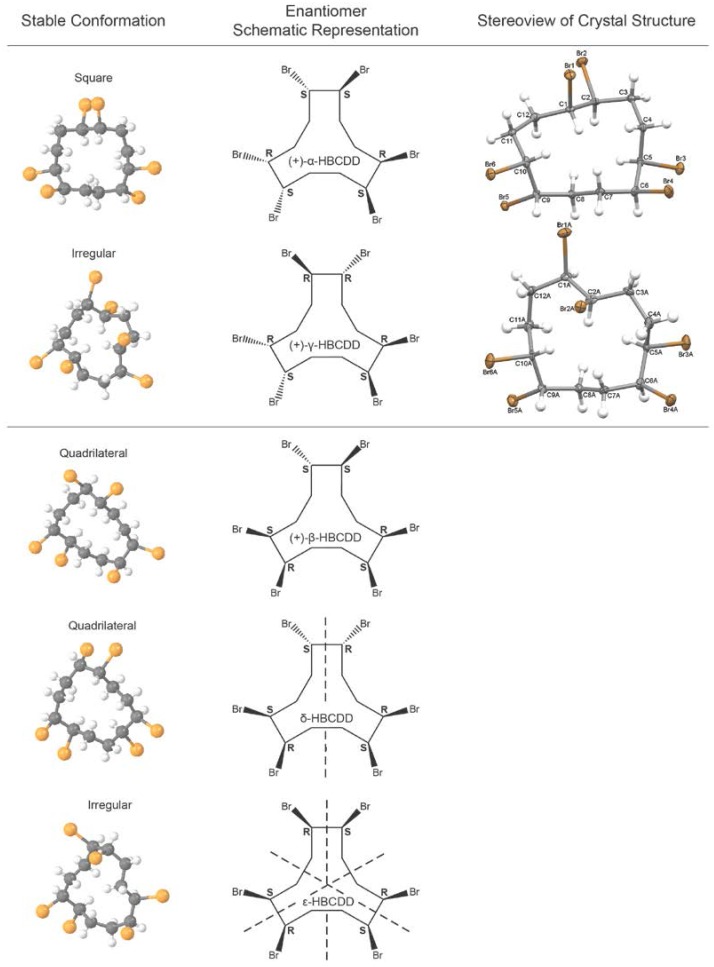
Stable conformations, schematic representations, and stereoviews of the crystal structures (when acquired) of enantiomerically pure (+)-α-HBCDD, (+)-γ-HBCDD, and (+)-β-HBCDD as well as the two minor isomers δ-HBCDD and ε-HBCDD.

Colourless crystals suitable for X-ray crystallography were able to be grown for the second eluting pSFC stereoisomers of α- and γ-HBCDD. The crystallizations were performed at room temperature and protected from light. Crystallization conditions were specifically chosen to avoid compromising the HBCDD samples through thermal rearrangement, debromination, or both, and the chemical purity of the bulk material (from which the isolated crystals for X-ray analysis were taken) was confirmed to be >98% by HRGC/LRMS, LCMS, and pSFC-MS. The resulting solid-state structures of these stereoisomers were found to be the α-1*S*,2*S*,5*R*,6*S*,9*S*,10*R*- and γ-1*R*,2*R*,5*R*,6*S*,9*S*,10*R*-enantiomers, respectively (Figure 1; see Supplementary Materials for more details including Flack parameters which confirm that the absolute configurations given by the structure refinements are correct), which correspond to (+)-α- and (+)-γ-HBCDD reported by Koeppen et al. Unfortunately, suitable crystals for X-ray structure determination could not be isolated for either of the β-HBCDD enantiomers. With the identity of the α- and γ-HBCDD fractions confirmed, samples were analyzed using the LC separation method described by Koeppen et al. in order to verify the published LC elution order and assign the configuration of the β enantiomers. To do this, samples of the isolated HBCDD enantiomers were first accurately weighed and dissolved in toluene at a concentration of 50 µg/mL, and stock solutions were diluted to a working concentration of 5 µg/mL using methanol. An accurate solution of technical HBCDD was also prepared and run in order to directly compare the chromatographic separation achieved with that originally reported. The LC elution order reported by Koeppen et al. of the (+)- and (−)-stereoisomers of α- and γ-HBCDD was confirmed, and the first eluting pSFC β-HBCDD enantiomer was identified as β-1*R*,2*R*,5*S*,6*R*,9*S*,10*R*-HBCDD, also known as the (−)-β-HBCDD enantiomer.

### 2.3. Enantiomer Resolution

**Figure 2:**
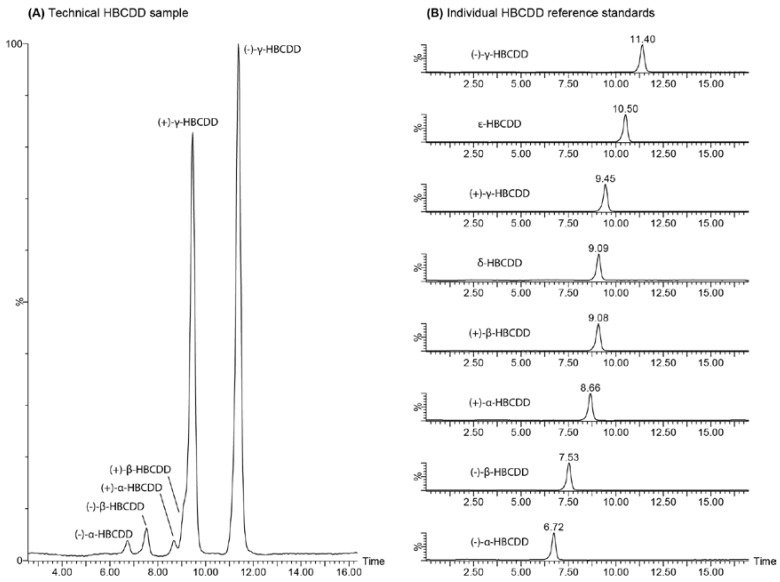
Analytical scale LC-MS separation of (**A**) a technical HBCDD mixture and (**B**) the isolated α-, β-, and γ-HBCDD enantiomers as well as reference standards for δ- and ε-HBCDD on a Phenomenex Nucleodex β-PM (permethylated β-cyclodextrin) stationary phase at ambient temperature using an acetonitrile/water mobile phase.

All of the isolated HBCDD enantiomer fractions were analyzed by LC-MS to confirm enantiomeric purity using a Nucleodex β-PM chiral analytical column, which is commonly cited in enantioselective liquid chromatography separations of HBCDD [16,22,32,33]. Since Koeppen et al. also utilized this column to separate a technical mixture of HBCDD [32] in order to demonstrate the effectiveness of this stationary phase, it was determined that their published gradient elution would be employed for comparison with the described pSFC separation. In addition to the isolated α-, β-, and γ-HBCDD enantiomers (the main components of technical HBCDD mixtures), two other diastereomers of HBCDD reported to be present in technical HBCDD products (δ- and ε-HBCDD) [34] were also investigated. These meso forms of HBCDD are achiral and therefore will not have enantiomers. However, since they are present in the technical product and have also been reported in both abiotic and biotic matrices [4], their elution relative to the α-, β-, and γ-HBCDD enantiomers is important to consider when evaluating a chiral HBCDD separation technique. Figure 2 illustrates the LC separation achieved using the Nucleodex β-PM chiral stationary phase. Although the (+)-β-HBCDD appears as a shoulder on the (+)-γ-HBCDD peak in the technical HBCDD mixture analyzed, the separation is very similar to that reported by Koeppen et al. Of substantial significance is the co-elution observed between δ-HBCDD and (+)-β-HBCDD on this stationary phase. If not properly resolved during an enantioselective analysis of environmental or biological samples, the presence of δ-HBCDD could artificially inflate the area of this β-HBCDD enantiomer, causing the enantiomeric fraction value to falsely deviate from 0.5.

**Figure 3:**
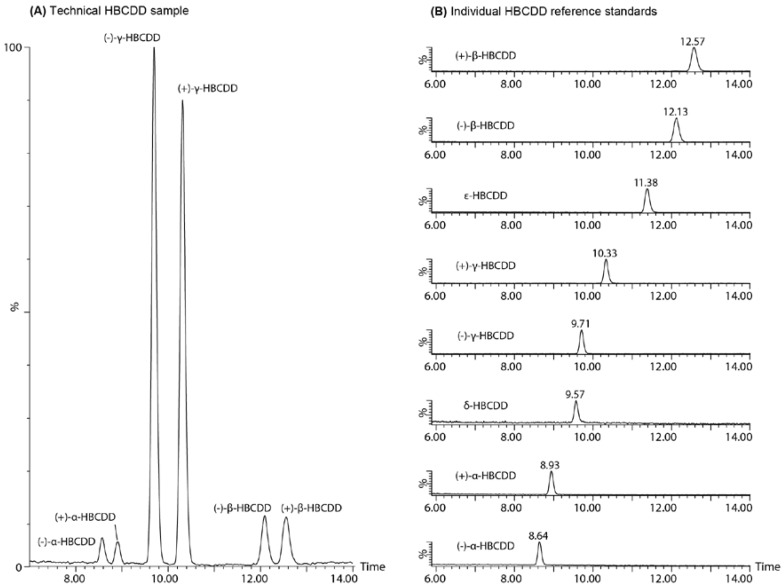
Analytical scale pSFC-MS separation of (**A**) a technical HBCDD mixture and (**B**) the isolated α-, β-, and γ-HBCDD enantiomers as well as reference standards for δ- and ε-HBCDD on a Trefoil CEL2 [cellulose tris-(3-chloro-4-methylphenylcarbamate)] stationary phase at 50 °C using an isopropanol modified carbon dioxide mobile phase.

In comparison, Figure 3 shows the same HBCDD samples run on the Trefoil CEL2 [cellulose tris-(3-chloro-4-methylphenylcarbamate)] stationary phase using the described analytical pSFC separation method. Of interest is the change in elution order from the traditional chiral LC separation. That is, (−)-α-HBCDD elutes first during the chiral LC separation followed by (−)-β-, (+)-α-, (+)-β-, δ-, (+)-γ-, ε-, and finally (−)-γ-HBCDD, but the elution order changes to (−)-α-, (+)-α-, δ-, (−)-γ-, (+)-γ-, ε-, (−)-β-, and finally (+)-β-HBCDD by pSFC. Although the overall elution order is very different, if the enantiomer pairs are examined specifically, only the (+)/(−)-γ-HBCDD enantiomer pair changes order. Additionally, baseline separation of all enantiomers was achieved, and both δ- and ε-HBCDD demonstrated adequate resolution from their closest neighbour (10% valley between δ-HBCDD and (−)-γ-HBCDD and baseline resolution between ε-HBCDD and (+)-β-HBCDD).

### 2.4. Chromatographic Considerations

Method development associated with pSFC separations, on both analytical and preparative scales, routinely involves the screening of multiple stationary phases and cosolvents with temperature, pressure, and gradient parameters that also require extensive optimization. This often entails an empirical approach; however, in chiral separations, the chemo- and enantio-selectivity of the chiral stationary phase can play an important role in the effectiveness of the separation. It has been reported that the chiral recognition mechanisms occurring during pSFC are similar to those taking place in LC and that one method does not provide superior enantio-resolution over the other [35]. However, certain CSPs can be considered better suited for a specific separation technique due to the chemical properties of the mobile phase and the mechanism of separation. For instance, it has been reported that the formation of inclusion complexes between cyclodextrin CSPs and chiral analytes can be limited by the low polarity of the carbon dioxide mobile phase in pSFC [36]. On the other hand, chiral polysaccharide-based stationary phases have been widely used for pSFC enantioseparations due to the broad enantioselectivity that has been demonstrated for this CSP and separation technique [37]. Indeed, cellulose and amylose CSPs are reported to be effective CSPs in pSFC based on their chiral recognition ability [38] and were therefore selected for investigation in this study (CEL1, CEL2, and AMY1, specifically). The ester and carbamate functional groups of these CSPs are likely to be the predominant sites of interaction with the chiral analyte. Hydrogen bonding is expected to dominate the retention mechanism associated with the HBCDD enantiomers; however, a multitude of weaker intermolecular interactions may contribute to complexation (e.g., halogen bonds) [39]. The substituents present on the phenyl moieties of polysaccharide phenylcarbamates modify the chemoselectivity of the CSP by altering the electron density associated with the carbamate; thus, its interaction with different analytes. It has been suggested that electron-withdrawing substituents increase the acidity of the carbamate N–H proton, while electron-donating substituents increase the electron density of the carbamate carbonyl oxygen [38]. Interaction between the CSP and HBCDD stereoisomers will depend on both the gross molecular shape of the chiral analytes as well as the accessibility of bromine atoms to facilitate transient bonding. HBCDD isomers can be grouped by their most stable conformations (determined by X-ray structure determination), with α being classified as “square”, β and δ as “quadrilateral”, and γ as ε “irregular” (with major distortion) (see Figure 1). The syn kinks present in the square (α-HBCDD) and quadrilateral (β-HBCDD and δ-HBCDD) conformations minimize unfavourable intramolecular interactions. The higher energy irregular conformations of γ-HBCDD and ε-HBCDD are also required to minimize intramolecular interactions due to unfavourable Br–Br and Br–C interactions. The orientation of the bromine atoms in these stable conformations and their subsequent availability for hydrogen bonding (presumably to the N–H of the CSP carbamate) may then be a major factor in dictating the retention of each isomer. Since enantiodiscrimination requires a three-point interaction [35] between the CSP and the HBCDD analyte, the molecular shape of the HBCDD stereoisomer is an important factor in its retention. Stereoisomers with similar conformations should exhibit close retention times on the polysaccharide CSPs since they have similar probabilities of complexation, but this is complicated by the inherent flexibility of their cyclododecane ring structures. Amylose CSPs are known to exhibit lower conformational stability resulting in conformational isomers with less effective hydrogen bonding sites; this could explain the poor isomeric resolution observed for this CSP. On the other hand, the uniformity of the cellulose CSPs may promote interaction and hence provide superior separation. It should also be noted that temperature-dependent reversal of enantiomer elution order is a well-known phenomenon on polysaccharide-based CSPs [38]; therefore, the pSFC elution order presented in this work should be associated with the stated temperature parameters.

## 3. Materials and Methods

### 3.1. Chemicals

All certified reference standards including alpha (α)-, beta (β)-, gamma (γ)-, delta (δ)-, and epsilon (ε)-1,2,5,6,9,10-hexabromocyclododecane (HBCDD) (50 μg/mL in toluene; >98% chemical purity) as well as crystalline samples of α-, β-, and γ-HBCDD for enantiomeric separation were obtained from Wellington Laboratories Inc. (Guelph, ON, Canada). Technical grade HBCDD (3N-BR-641, lot: 3N-06303) was obtained from 3N International Inc. (Akron, OH, USA). HPLC grade methanol, water, and acetonitrile as well as distilled in glass grade dichloromethane were purchased from Caledon (Guelph, ON, Canada). LC-MS Chromasolv grade 2-propanol and *n*-heptane, as well as inhibitor-free anhydrous cyclopentyl methyl ether, were purchased from Sigma-Aldrich (Oakville, ON, Canada). HPLC grade ethyl acetate was purchased from Fisher Scientific (Ottawa, ON, Canada). Denatured ethyl alcohol was purchased from Fisher Scientific (Fair Lawn, NJ, USA). Food grade carbon dioxide was purchased from Linde Canada Industrial Gases (Guelph, ON, Canada), and research grade helium (99.9999%) was purchased from Praxair Canada Inc. (Mississauga, ON, Canada).

### 3.2. Chromatographic Systems and Conditions

#### 3.2.1. Isolation of Enantiomerically Pure (+)- and (−)-α-, β-, and γ-HBCDD by Preparative-Scale pSFC

Preparative-scale pSFC separations were carried out using a Waters Prep15 SFC system (Waters Corp., Milford, MA, USA) coupled with a Waters 3100 benchtop single quadrupole mass detector (Waters Corp., Milford, MA, USA) configured in negative ion atmospheric pressure chemical ionization (APCI) mode with the following parameters: corona needle (uA) = 5.00, cone voltage (V) = 30.00; desolvation gas flow (L/h) = 550; desolvation temperature (°C) = 350; source temperature (°C) = 125. Methanol was used as the MS make-up solvent and was added to the split from the Prep15 system at a flow rate of 1 mL/min. Pseudomolecular ion clusters corresponding to [M − H]ˉ were generated with these settings for all HBCDD isomers in full scan mode (*m*/*z* 400–800), but the MS data were used for confirmatory purposes only. Timed collection windows were determined using the PDA data (UV absorbance at 225 nm) due to relative strength of this signal compared with that generated using full scan MS (see Figures S1–S3). Resolution of the enantiomers of α- and γ-HBCDD was accomplished on a Phenomenex Lux Cellulose-2 column ([cellulose tris(3-chloro-4-methylphenylcarbamate)]; 5.0 µm, 10 × 250 mm) at 40 °C. The α-HBCDD enantiomers were separated using a 2-propanol modified carbon dioxide mobile phase with the back pressure maintained at 1450 psi at a flow rate of 15 mL/min. The elution program involved an isocratic hold at 13% cosolvent for 16.5 min before being quickly ramped (over 30 s) to 20% cosolvent and held for 5 min. The gradient was then returned to initial conditions for a total run time of 23 min. Alternatively, the following isocratic method was utilized for the preparatory separation and collection of the γ-HBCDD enantiomers: 20% 2-propanol modified carbon dioxide mobile phase with the back pressure maintained at 1740 psi, a flow rate of 15 mL/min, and a total run time of 14 min. Optimal separations of the enantiomers of β-HBCDD were carried out on a Phenomenex Lux Cellulose-1 column ([cellulose tris(3,5-dimethylphenylcarbamate)]; 5.0 µm, 10 × 250 mm) at 40 °C using an isocratic 15% acetonitrile modified carbon dioxide mobile phase with the back pressure maintained at 1595 psi, a flow rate of 15 mL/min, and a total run time of 17 min. All samples were injected at a concentration of 5 mg/mL in 50:50 methanol:cyclopentyl methyl ether.

All HBCDD enantiomers were collected using timed fraction collection, and methanol was used as the gas liquid separator (GLS) make-up solvent with a flow rate of 4 mL/min. Under these conditions, no issues with sample solubility or carry-over were experienced. The resulting fractions were rotary evaporated to dryness under reduced pressure.

#### 3.2.2. Analytical pSFC-MS Analysis of the HBCDD Enantiomers

Analytical-scale pSFC separations of all purified HBCDD enantiomer samples as well as technical HBCDD material were carried out using a Waters Acquity Ultra Performance Convergence Chromatography (UPC^2^) (Waters Corp., Milford, MA, USA) system. The UPC^2^ was coupled with a Micromass Quattro micro atmospheric pressure ionization (API) Mass Spectrometer (MS) (Waters Corp., Milford, MA, USA) configured in negative-ion electrospray ionization (ESI) mode with the following parameters: capillary voltage (kV) = 3.00, cone voltage (V) = 18.00; cone gas flow (L/h) = 50; desolvation gas flow (L/h) = 550; desolvation temperature (°C) = 350; source temperature (°C) = 120. Methanol was used as the MS make-up solvent and was added to the split from the UPC^2^ at a flow rate of 0.35 mL/min. Pseudomolecular ion clusters corresponding to [M − H]ˉ were generated with these settings for all HBCDD isomers. MS data were acquired in SIR mode, with a dwell time of 0.1 s for each mass, and processed using Waters MassLynx software. Optimal separations were achieved with a Waters Trefoil CEL2 column ([cellulose tris-(3-chloro-4-methylphenylcarbamate)]; 2.5 μm, 3.0 × 150 mm) at 50 °C using a 2-propanol modified carbon dioxide (CO_2_) mobile phase, with the back pressure maintained at 2000 psi at a flow rate of 1 mL/min. In order to eliminate carry-over between injections, weak and strong solvent washes consisting of 80:20 cyclohexane:2-propanol and 50:50 cyclohexane:ethyl acetate, respectively, were utilized. The gradient elution program was also split into three injections consisting of the sample run, followed by a short injector flush with toluene, and a re-equilibration run. Initial conditions for the separation run were 95% CO_2_, 5% 2-propanol followed by a ramp to 25% 2-propanol over 10 min and a subsequent 4 min hold (run time = 14 min). The injector flush consisted of the injection of a toluene blank into isocratic conditions of 75% CO_2_ and 25% 2-propanol (run time = 3.00 min). Finally, the re-equilibration run started at 75% CO_2_ and 25% 2-propanol, and was held for 1.75 min before being ramped to 95% CO_2_ and 5% 2-propanol over 0.50 min (run time = 3.00 min). The combined run time for a single sample was 20 min. All HBCDD samples were prepared for pSFC-MS analysis on an analytical scale through dilution of toluene stock solutions to a concentration of 5 µg/mL using 80:20 *n*-heptane:2-propanol.

#### 3.2.3. LC-MS Analysis of the HBCDD Enantiomers

LC-MS analyses of all HBCDD samples were conducted using a Waters Acquity Ultra Performance Liquid Chromatography (UPLC) System (Waters Corp., Milford, MA, USA) coupled with the same Micromass Quattro micro API MS (Waters Corp., Milford, MA, USA) listed above. For LC analyses, the MS was configured in negative-ion electrospray ionization (ESI) mode with the following parameters: capillary voltage (kV) = 2.00, cone voltage (V) = 18.00; cone gas flow (L/h) = 65; desolvation gas flow (L/h) = 750; desolvation temperature (°C) = 350; source temperature (°C) = 100. Pseudomolecular ion clusters corresponding to [M − H]ˉ were generated with these settings for all HBCDD isomers. MS data were acquired in full scan and MRM modes with a dwell time of 0.1 s for each transition and processed using Waters MassLynx software. Optimal enantioseparations were achieved with a Phenomenex Nucleodex β-PM column (permethylated β-cyclodextrin; 5 μm, 4.6 × 200 mm) at ambient temperature using the following acetonitrile and water gradient elution at 1 mL/min: initial conditions of 60% acetonitrile and 40% water were ramped to 85% acetonitrile over 10 min followed by a second ramp to 90% acetonitrile over 2 min. After a 3 min hold at 90% acetonitrile, the gradient was returned to initial conditions (60% acetonitrile) over 1 min for a total run time of 17.00 min.

#### 3.2.4. X-ray Diffraction Studies

Single crystals of suitable quality for X-ray diffraction studies were obtained at room temperature for the second eluting stereoisomers of α- and γ-HBCDD that were isolated from preparative scale pSFC. Crystals were grown by slow evaporation of a small volume and low concentration solution of the bulk material. Dichloromethane was used as the crystallizing solvent for the alpha isomer and ethyl acetate was used as the crystallizing solvent for the gamma isomer. Single crystal X-ray diffraction data for (+)-α-HBCDD and (+)-γ-HBCDD were collected on a Bruker Kappa APEX-DUO CCD using Mo Kα radiation (λ = 0.71073 Å) at a temperature of 147(2) K. Structures were solved and refined using SHELXL-2014/7. CCDC 1509809-1509810 contains the crystallographic data for this paper. These data can be obtained free of charge via http://www.ccdc.cam.ac.uk/conts/retrieving.html (or from the CCDC, 12 Union Road, Cambridge CB2 1EZ, UK; Fax: +44 1223 336033; E-mail: deposit@ccdc.cam.ac.uk).

In order to verify that the isolated material from which the crystals were grown was chemically pure, all samples sent for X-ray crystallography were run by high-resolution gas chromatography (HRGC) coupled with a low-resolution mass spectrometer (LRMS) in addition to LC-MS and pSFC-MS analysis. HRGC/LRMS analyses were conducted on an Agilent 7890A Gas Chromatograph/5975C MSD (Agilent Technologies, Santa Clara, CA, USA) and chromatographic separations were carried out using an Agilent J&W DB5 (30 m × 0.25 mm ID, 0.25 µm film thickness) column. All injections were 1 µL at a temperature of 200 °C in splitless injection mode and data were acquired in full scan mode (*m*/*z* 50–1000). The following temperature program was utilized: initial oven temperature 140 °C, hold for 1 min, ramp at 40 °C/min to 200 °C, hold for 5.5 min, ramp at 10.0 °C/min to 325 °C, hold for 20 min (total run time 40.5 min).

## 4. Conclusions

The six main stereoisomers of technical HBCDD (i.e., the (+)-α-, (−)-α-, (+)-β-, (−)-β-, (+)-γ-, and (−)-γ- enantiomers of HBCDD) were separated and isolated using enantioselective packed column supercritical fluid chromatography (pSFC) separation methods on a preparative scale. Characterization was completed using published chiral liquid chromatography (LC) methods and elution profiles, as well as X-ray crystallography, and the isolated fractions were definitively identified. A comparison of the resolution achieved for the HBCDD enantiomers along with two meso isomers (δ- and ε-HBCDD) reported to be present in technical samples was also investigated on an analytical scale using both LC and pSFC. Changes in elution order were highlighted with the most notable variation between techniques being the elution order of the (+)/(−)-γ-HBCDD enantiomer pair. Using the pSFC method described herein on a CEL2 [cellulose tris-(3-chloro-4-methylphenylcarbamate)] chiral stationary phase, the elution order of HBCDD stereoisomers known to be present in technical HBCDD mixtures proceeds as follows (from first eluter to last eluter): (−)-α-, (+)-α-, δ-, (−)-γ-, (+)-γ-, ε-, (−)-β-, and (+)-β-HBCDD. The successful application of a green enantioselective separation technique (chiral pSFC) to an environmental contaminant of concern emphasizes the potential associated with this method for analyzing and isolating similar compounds.

## Supplementary Materials

Supplementary materials can be accessed at: http://www.mdpi.com/1420-3049/21/11/1509/s1: Figure S1: Chromatogram illustrating the separation of α-HBCDD enantiomers achieved during preparatory separation using the described method; Figure S2: Chromatogram illustrating the separation of β-HBCDD enantiomers achieved during preparatory separation using the described method; Figure S3: Chromatogram illustrating the separation of γ-HBCDD enantiomers achieved during preparatory separation using the described method; Table S1: Crystal data and structure refinement for (+)-α-HBCDD; Table S2: Atomic coordinates (×10^4^) and equivalent isotropic displacement parameters (Å^2^x 10^3^) for (+)-α-HBCDD. U(eq) is defined as one third of the trace of the orthogonalized U^ij^ tensor; Table S3: Bond lengths [Å] and angles [°] for (+)-α-HBCDD; Table S4: Anisotropic displacement parameters (Å^2^x 10^3^) for (+)-α-HBCDD. The anisotropic displacement factor exponent takes the form: −2π^2^[ h^2^ a^*2^U^11^ + ... + 2 h k a* b* U^12^]; Table S5: Hydrogen coordinates (×10^4^) and isotropic displacement parameters (Å^2^x 10^3^) for (+)-α-HBCDD; Table S6: Torsion angles [°] for (+)-α-HBCDD; Table S7: Crystal data and structure refinement for (+)-γ-HBCDD; Table S8: Atomic coordinates (×10^4^) and equivalent isotropic displacement parameters (Å^2^x 10^3^) for (+)-γ-HBCDD. U(eq) is defined as one third of the trace of the orthogonalized U^ij^ tensor; Table S9: Bond lengths [Å] and angles [°] for (+)-γ-HBCDD; Table S10: Anisotropic displacement parameters (Å^2^x 10^3^) for (+)-γ-HBCDD. The anisotropic displacement factor exponent takes the form: −2π^2^[h^2^ a^*2^U^11^ + ... + 2 h k a* b* U^12^]; Table S11: Hydrogen coordinates (×10^4^) and isotropic displacement parameters (Å^2^x 10^3^) for (+)-γ-HBCDD; Table S12: Torsion angles [°] for (+)-γ-HBCDD.

## References

[1] Alaee M., Arias P., Sjodin A., Bergman A. (2003). An overview of commercially used brominated flame retardants, their applications, their use patterns in different countries/regions and possible modes of release. Environ. Int..

[2] Covaci A., Gerecke A.C., Law R.J., Voorspoels S., Kohler M., Heeb N.V., Leslie H., Allchin C.R., de Boer J. (2006). Hexabromocyclododecanes (HBCDs) in the environment and humans: A review. Environ. Sci. Technol..

[3] Hunziker R.W., Gonsior S., MacGregor J.A., Desjardins D., Ariano J., Friederich U. (2004). Fate and effect of hexabromocyclododecane in the environment. Organohalog. Compd..

[4] Koch C., Schmidt-Koetters T., Rupp R., Sures B. (2015). Review of hexabromocyclododecane (HBCD) with a focus on legislation and recent publications concerning toxicokinetics and -dynamics. Environ. Pollut..

[5] Law R.J., Covaci A., Harrad S., Herzke D., Abdallah M.A.E., Femie K., Toms L.-M.L., Takigami H. (2014). Levels and trends of PBDEs and HBCDs in the global environment: Status at the end of 2012. Environ. Int..

[6] Heeb N.V., Graf H., Schweizer W.B., Lienemann P. (2010). Thermally-induced transformation of hexabromocyclo dodecanes and isobutoxypenta bromocyclododecanes in flame-proofed polystyrene materials. Chemosphere.

[7] Harrad S., Abdallah M.A.E., Covaci A. (2009). Causes of variability in concentrations and diastereomer patterns of hexabromocyclododecanes in indoor dust. Environ. Int..

[8] Vorkamp K., Bester K., Riget F.F. (2012). Species-Specific Time Trends and Enantiomer Fractions of Hexabromocyclododecane (HBCD) in Biota from East Greenland. Environ. Sci. Technol..

[9] Borga K., Bidleman T.F. (2005). Enantiomer fractions of organic chlorinated pesticides in arctic marine lee fauna, zooplankton, and benthos. Environ. Sci. Technol..

[10] Hong H., Li D., Shen R., Wang X., Shi D. (2014). Mechanisms of hexabromocyclododecanes induced developmental toxicity in marine medaka (Oryzias melastigma) embryos. Aquat. Toxicol..

[11] Zhang X., Yang F., Xu C., Liu W., Wen S., Xu Y. (2008). Cytotoxicity evaluation of three pairs of hexabromocyclododecane (HBCD) enantiomers on Hep G2 cell. Toxicol. In Vitro.

[12] Esslinger S., Becker R., Jung C., Schroeter-Kermani C., Bremser W., Nehls I. (2011). Temporal trend (1988–2008) of hexabromocyclododecane enantiomers in herring gull eggs from the german coastal region. Chemosphere.

[13] He M.-J., Luo X.-J., Yu L.-H., Liu J., Zhang X.-L., Chen S.-J., Chen D., Mai B.-X. (2010). Tetrabromobisphenol-A and Hexabromocyclododecane in Birds from an E-Waste Region in South China: Influence of Diet on Diastereoisomer- and Enantiomer-Specific Distribution and Trophodynamics. Environ. Sci. Technol..

[14] Koeppen R., Becker R., Esslinger S., Nehls I. (2010). Enantiomer-specific analysis of hexabromocyclododecane in fish from Etnefjorden (Norway). Chemosphere.

[15] Sun Y.-X., Luo X.-J., Mo L., He M.-J., Zhang Q., Chen S.-J., Zou F.-S., Mai B.-X. (2012). Hexabromocyclododecane in terrestrial passerine birds from e-waste, urban and rural locations in the Pearl River Delta, South China: Levels, biomagnification, diastereoisomer- and enantiomer-specific accumulation. Environ. Pollut..

[16] Janak K., Covaci A., Voorspoels S., Becher G. (2005). Hexabromocyclododecane in marine species from the Western Scheldt Estuary: Diastereoisomer- and enantiomer-specific accumulation. Environ. Sci. Technol..

[17] Tomy G.T., Pleskach K., Oswald T., Halldorson T., Helm P.A., Macinnis G., Marvin C.H. (2008). Enantioselective bioaccumulation of hexabromocyclododecane and congener-specific accumulation of brominated diphenyl ethers in an eastern Canadian Arctic marine food web. Environ. Sci. Technol..

[18] Zhang X., Yang F., Luo C., Wen S., Zhang X., Xu Y. (2009). Bioaccumulative characteristics of hexabromocyclododecanes in freshwater species from an electronic waste recycling area in China. Chemosphere.

[19] Du M., Lin L., Yan C., Zhang X. (2012). Diastereoisomer- and Enantiomer-Specific Accumulation, Depuration, and Bioisomerization of Hexabromocyclododecanes in Zebrafish (Danio rerio). Environ. Sci. Technol..

[20] Zhang Y., Sun H., Ruan Y. (2014). Enantiomer-specific accumulation, depuration, metabolization and isomerization of hexabromocyclododecane (HBCD) diastereomers in mirror carp from water. J. Hazard. Mater..

[21] Abdallah M.A.-E., Uchea C., Chipman J.K., Harrad S. (2014). Enantioselective Biotransformation of Hexabromocyclododecane by in Vitro Rat and Trout Hepatic Sub-Cellular Fractions. Environ. Sci. Technol..

[22] Heeb N.V., Schweizer W.B., Mattrel P., Haag R., Gerecke A.C., Schmid P., Zennegg M., Vonmont H. (2008). Regio- and stereoselective isomerization of hexabromocyclododecanes (HBCDs): Kinetics and mechanism of gamma- to alpha-HBCD isomerization. Chemosphere.

[23] Esslinger S., Becker R., Mueller-Belecke A., Bremser W., Iung C., Nehls I. (2010). HBCD Stereoisomer Pattern in Mirror Carps Following Dietary Exposure to Pure gamma-HBCD Enantiomers. J. Agric. Food Chem..

[24] Esslinger S., Becker R., Maul R., Nehls I. (2011). Hexabromocyclododecane Enantiomers: Microsomal Degradation and Patterns of Hydroxylated Metabolites. Environ. Sci. Technol..

[25] Guerra P., Eliarrat E., Barcelo D. (2008). Enantiomeric specific determination of hexabromocyclododecane by liquid chromatography-quadrupole linear ion trap mass spectrometry in sediment samples. J. Chromatogr. A.

[26] Marvin C.H., MacInnis G., Alaee M., Arsenault G., Tomy G.T. (2007). Factors influencing enantiomeric fractions of hexabromocyclododecane measured using liquid chromatography/tandem mass spectrometry. Rapid Commun. Mass Spectrom..

[27] Dodder N.G., Peck A.M., Kucklick J.R., Sander L.C. (2006). Analysis of hexabromocyclododecane diastereomers and enantiomers by liquid chromatography/tandem mass spectrometry: Chromatographic selectivity and ionization matrix effects. J. Chromatogr. A.

[28] Bester K., Vorkamp K. (2013). A two-dimensional HPLC separation for the enantioselective determination of hexabromocyclododecane (HBCD) isomers in biota samples. Anal. Bioanal. Chem..

[29] Mullin L., Burgess J.A., Jogsten I.E., Geng D., Aubin A., van Bavel B. (2015). Rapid separation of hexabromocyclododecane diastereomers using a novel method combining convergence chromatography and tandem mass spectrometry. Anal. Methods.

[30] Vanwasen U., Swaid I., Schneider G.M. (1980). Physicochemical Principles and Applications of Supercritical Fluid Chromatography (SFC). Angew. Chem. Int. Ed..

[31] Morita A., Kajimoto O. (1990). Solute Solvent Interaction in Nonpolar Supercritical Fluid—A Clustering Model and Size Distribution. J. Phys. Chem..

[32] Koeppen R., Becker R., Emmerling F., Jung C., Nehls I. (2007). Enantioselective preparative HPLC separation of the HBCD—Stereoisomers from the technical product and their absolute structure elucidation using X-ray crystallography. Chirality.

[33] Heeb N.V., Schweizer W.B., Mattrel P., Haag R., Gerecke A.C., Kohler M., Schmid P., Zennegg M., Wolfensberger M. (2007). Solid-state conformations and absolute configurations of (+) and (-)alpha-, beta-, and gamma-hexabromocyclododecanes (HBCDs). Chemosphere.

[34] Arsenault G., Konstantinov A., Marvin C.H., MacInnis G., McAlees A., McCrindle R., Riddell N., Tomy G.T., Yeo B. (2007). Synthesis of the two minor isomers, delta- and epsilon-1,2,5,6,9,10-hexabromocyclododecane, present in commercial hexabromocyclododecane. Chemosphere.

[35] De Klerck K., Mangelings D., Heyden Y.V. (2012). Supercritical fluid chromatography for the enantioseparation of pharmaceuticals. J. Pharm. Biomed. Anal..

[36] Xiao Y., Ng S.C., Tan T.T.Y., Wang Y. (2012). Recent development of cyclodextrin chiral stationary phases and their applications in chromatography. J. Chromatogr. A.

[37] Da Silva C.G.A., Collins C.H. (2014). Super/Subcritical Fluid Chromatography with Packed Columns: State of the Art and Applications. Quim. Nova.

[38] Chankvetadze B. (2012). Recent developments on polysaccharide-based chiral stationary phases for liquid-phase separation of enantiomers. J. Chromatogr. A.

[39] Bissantz C., Kuhn B., Stahl M. (2010). A Medicinal Chemist’s Guide to Molecular Interactions. J. Med. Chem..

