# Supplementary material for: Enantioselective Analytical- and Preparative-Scale Separation of Hexabromocyclododecane Stereoisomers Using Packed Column Supercritical Fluid Chromatography
Source: Molecules. 2016 Nov 10;21(11):1509. doi: 10.3390/molecules21111509 (PMC6273052; doi:10.3390/molecules21111509)
Supplement: Supplementary file 1 [file molecules-21-01509-s001.pdf]

Supplementary Materials: Enantioselective Analytical- and Preparative-Scale Separation of Hexabromocyclododecane Stereoisomers Using Packed Column Supercritical Fluid Chromatography

Nicole Riddell, Lauren Gayle Mullin, Bert van Bavel, Ingrid Ericson Jogsten, Alan McAlees, Allison Brazeau, Scott Synnott, Alan Lough, Robert McCrindle and Brock Chittim

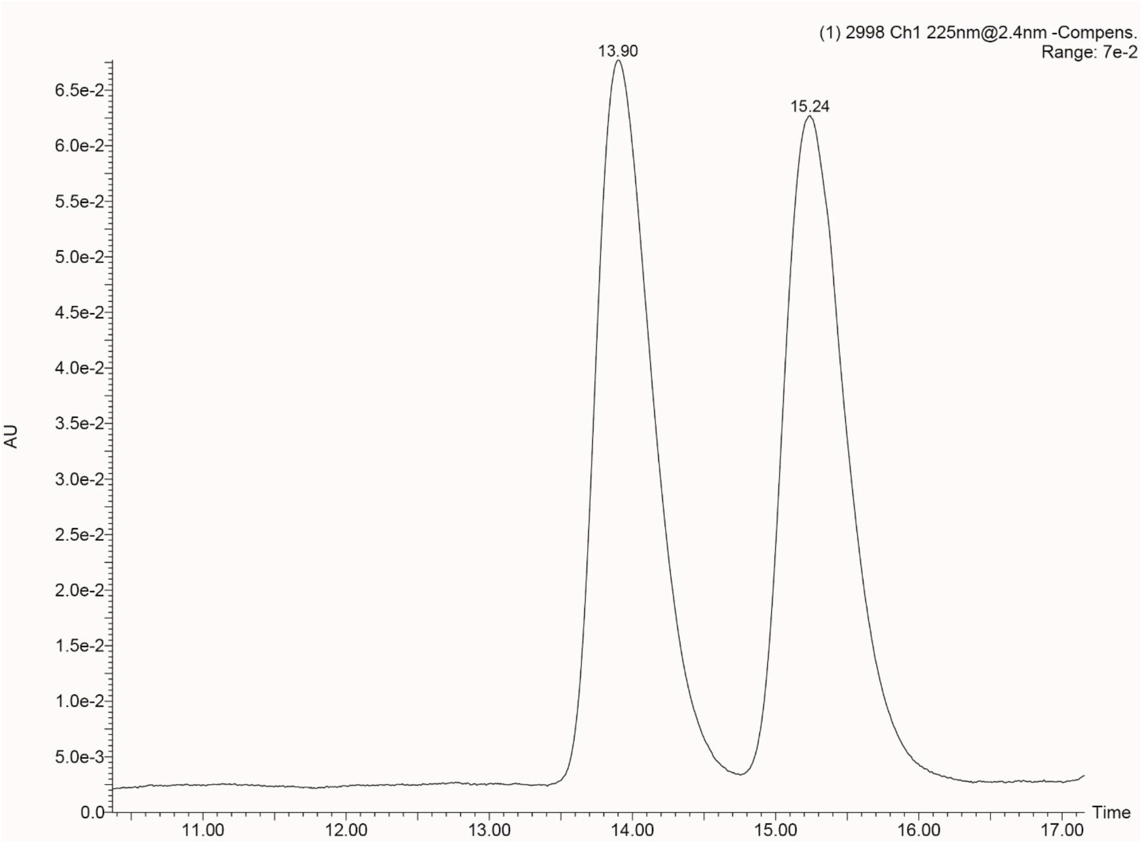

Figure S1. Chromatogram illustrating the separation of α-HBCDD enantiomers achieved during preparatory separation using the described method.

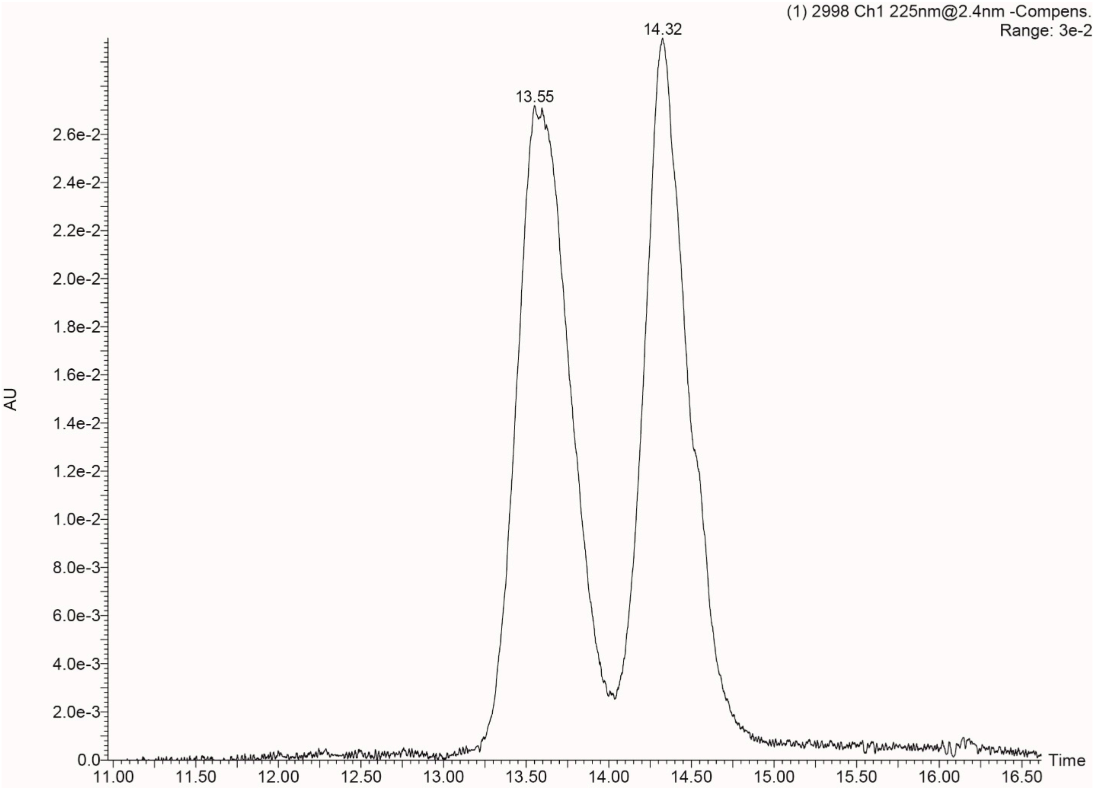

Figure S2. Chromatogram illustrating the separation of β-HBCDD enantiomers achieved during preparatory separation using the described method.

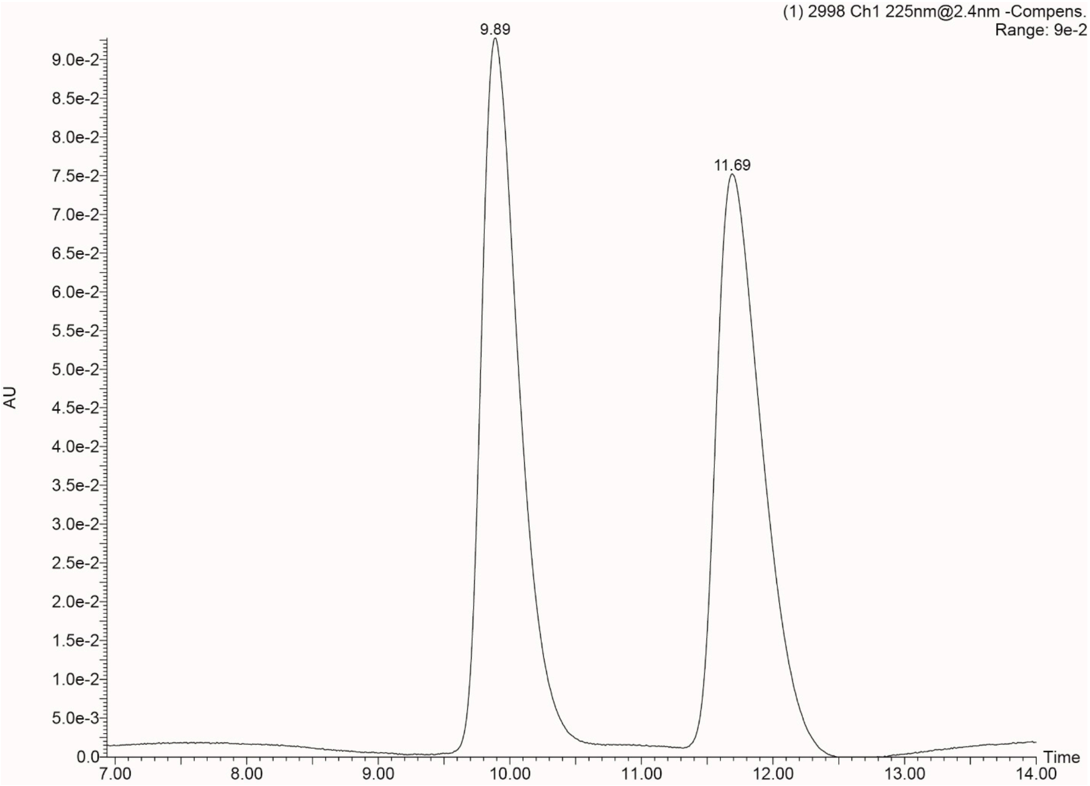

Figure S3. Chromatogram illustrating the separation of γ-HBCDD enantiomers achieved during preparatory separation using the described method.

Table S1. Crystal data and structure refinement for (+)-α-HBCDD.

**Empirical Formula**	**C_{12} H_{18} Br_6**
Formula weight	641.72
Temperature	147(2) K
Wavelength	0.71073 Å
Crystal system	Orthorhombic
Space group	$P2_12_12_1$
Unit cell dimensions	a = 7.1332(6) Å α= 90°.<br>b = 13.8007(13) Å β= 90°.<br>c = 17.5296(17) Å γ= 90°.
Volume	1725.7(3) Å^3
Z	4
Density (calculated)	2.470 Mg/m^3
Absorption coefficient	13.942 mm^{-1}
F(000)	1200
Crystal size	0.200 × 0.150 × 0.060 mm^3
Theta range for data collection	1.878 to 27.620°.
Index ranges	−9 ≤ h ≤ 8, −17 ≤ k ≤ 17, −22 ≤ l ≤ 22
Reflections collected	28,792
Independent reflections	3992 [R(int) = 0.0433]
Completeness to theta = 25.242°	100.0%
Absorption correction	Semi-empirical from equivalents
Max. and min. transmission	0.7456 and 0.3691
Refinement method	Full-matrix least-squares on F^2
Data/restraints/parameters	3992/0/163
Goodness-of-fit on F^2	1.032
Final R indices [I>2sigma(I)]	R1 = 0.0200, wR2 = 0.0314
R indices (all data)	R1 = 0.0248, wR2 = 0.0321
Absolute structure parameter	0.003(7)
Extinction coefficient	n/a
Largest diff. peak and hole	0.511 and −0.406 e.Å^{-3}
Flack parameter	0.003(7)

Table S2. Atomic coordinates (×10^4) and equivalent isotropic displacement parameters (Å^{2}x 10^3) for (+)-α-HBCDD. U(eq) is defined as one third of the trace of the orthogonalized U^{ij} tensor.

	**x**	**x**	**z**	**U(eq)**
Br(1)	8871(1)	4952(1)	6826(1)	19(1)
Br(2)	4218(1)	5724(1)	6306(1)	21(1)
Br(3)	2139(1)	4027(1)	9224(1)	20(1)
Br(4)	6698(1)	3331(1)	9968(1)	23(1)
Br(5)	7889(1)	8324(1)	9156(1)	15(1)
Br(6)	12525(1)	7277(1)	8680(1)	17(1)
C(1)	7114(5)	5689(3)	7462(2)	12(1)
C(2)	5094(5)	5388(3)	7337(2)	14(1)
C(3)	4680(5)	4321(3)	7502(2)	14(1)
C(4)	5413(5)	3986(3)	8276(2)	14(1)
C(5)	4620(5)	4556(3)	8953(2)	13(1)
C(6)	5855(5)	4624(3)	9656(2)	14(1)
C(7)	7585(5)	5269(2)	9532(2)	13(1)
C(8)	7077(5)	6324(2)	9341(2)	12(1)
C(9)	8789(5)	6967(2)	9204(2)	12(1)

C(10)	9985(5)	6735(3)	8499(2)	14(1)
C(11)	9420(5)	7090(3)	7702(2)	13(1)
C(12)	7488(5)	6773(3)	7409(2)	14(1)

Table S3. Bond lengths [Å] and angles [°] for (+)-α-HBCDD.

Br(1)-C(1)	1.962(3)
Br(2)-C(2)	1.967(4)
Br(3)-C(5)	1.973(4)
Br(4)-C(6)	1.961(3)
Br(5)-C(9)	1.981(3)
Br(6)-C(10)	1.985(4)
C(1)-C(2)	1.515(5)
C(1)-C(12)	1.522(5)
C(1)-H(1A)	1.0000
C(2)-C(3)	1.530(5)
C(2)-H(2A)	1.0000
C(3)-C(4)	1.526(5)
C(3)-H(3A)	0.9900
C(3)-H(3B)	0.9900
C(4)-C(5)	1.533(5)
C(4)-H(4A)	0.9900
C(4)-H(4B)	0.9900
C(5)-C(6)	1.517(5)
C(5)-H(5A)	1.0000
C(6)-C(7)	1.537(5)
C(6)-H(6A)	1.0000
C(7)-C(8)	1.538(5)
C(7)-H(7A)	0.9900
C(7)-H(7B)	0.9900
C(8)-C(9)	1.529(5)
C(8)-H(8A)	0.9900
C(8)-H(8B)	0.9900
C(9)-C(10)	1.536(5)
C(9)-H(9A)	1.0000
C(10)-C(11)	1.534(5)
C(10)-H(10A)	1.0000
C(11)-C(12)	1.534(5)
C(11)-H(11A)	0.9900
C(11)-H(11B)	0.9900
C(12)-H(12A)	0.9900
C(12)-H(12B)	0.9900
C(2)-C(1)-C(12)	115.3(3)
C(2)-C(1)-Br(1)	112.5(2)
C(12)-C(1)-Br(1)	111.3(2)
C(2)-C(1)-H(1A)	105.6
C(12)-C(1)-H(1A)	105.6
Br(1)-C(1)-H(1A)	105.6
C(1)-C(2)-C(3)	114.9(3)
C(1)-C(2)-Br(2)	111.7(3)
C(3)-C(2)-Br(2)	109.8(3)
C(1)-C(2)-H(2A)	106.7
C(3)-C(2)-H(2A)	106.7
Br(2)-C(2)-H(2A)	106.7
C(4)-C(3)-C(2)	113.1(3)
C(4)-C(3)-H(3A)	109.0
C(2)-C(3)-H(3A)	109.0
C(4)-C(3)-H(3B)	109.0

C(2)-C(3)-H(3B)	109.0
H(3A)-C(3)-H(3B)	107.8
C(3)-C(4)-C(5)	114.1(3)
C(3)-C(4)-H(4A)	108.7
C(5)-C(4)-H(4A)	108.7
C(3)-C(4)-H(4B)	108.7
C(5)-C(4)-H(4B)	108.7
H(4A)-C(4)-H(4B)	107.6
C(6)-C(5)-C(4)	116.5(3)
C(6)-C(5)-Br(3)	110.4(3)
C(4)-C(5)-Br(3)	109.1(2)
C(6)-C(5)-H(5A)	106.8
C(4)-C(5)-H(5A)	106.8
Br(3)-C(5)-H(5A)	106.8
C(5)-C(6)-C(7)	112.8(3)
C(5)-C(6)-Br(4)	110.4(3)
C(7)-C(6)-Br(4)	108.7(2)
C(5)-C(6)-H(6A)	108.3
C(7)-C(6)-H(6A)	108.3
Br(4)-C(6)-H(6A)	108.3
C(6)-C(7)-C(8)	112.9(3)
C(6)-C(7)-H(7A)	109.0
C(8)-C(7)-H(7A)	109.0
C(6)-C(7)-H(7B)	109.0
C(8)-C(7)-H(7B)	109.0
H(7A)-C(7)-H(7B)	107.8
C(9)-C(8)-C(7)	113.3(3)
C(9)-C(8)-H(8A)	108.9
C(7)-C(8)-H(8A)	108.9
C(9)-C(8)-H(8B)	108.9
C(7)-C(8)-H(8B)	108.9
H(8A)-C(8)-H(8B)	107.7
C(8)-C(9)-C(10)	116.7(3)
C(8)-C(9)-Br(5)	107.3(2)
C(10)-C(9)-Br(5)	110.0(2)
C(8)-C(9)-H(9A)	107.5
C(10)-C(9)-H(9A)	107.5
Br(5)-C(9)-H(9A)	107.5
C(11)-C(10)-C(9)	121.3(3)
C(11)-C(10)-Br(6)	105.4(2)
C(9)-C(10)-Br(6)	107.4(2)
C(11)-C(10)-H(10A)	107.3
C(9)-C(10)-H(10A)	107.3
Br(6)-C(10)-H(10A)	107.3
C(10)-C(11)-C(12)	116.8(3)
C(10)-C(11)-H(11A)	108.1
C(12)-C(11)-H(11A)	108.1
C(10)-C(11)-H(11B)	108.1
C(12)-C(11)-H(11B)	108.1
H(11A)-C(11)-H(11B)	107.3
C(1)-C(12)-C(11)	114.7(3)
C(1)-C(12)-H(12A)	108.6
C(11)-C(12)-H(12A)	108.6
C(1)-C(12)-H(12B)	108.6
C(11)-C(12)-H(12B)	108.6
H(12A)-C(12)-H(12B)	107.6

Symmetry transformations used to generate equivalent atoms.

Table S4. Anisotropic displacement parameters (Å²x 10^3) for (+)-α-HBCDD. The anisotropic displacement factor exponent takes the form: $-2\pi^2[h^2 a^{*2}U^{11} + ... + 2 h k a^* b^* U^{12}]$.

	U^{11}	U^{22}	U^{33}	U^{23}	U^{13}	U^{12}
Br(1)	17(1)	19(1)	22(1)	−4(1)	5(1)	3(1)
Br(2)	19(1)	30(1)	14(1)	5(1)	−4(1)	−3(1)
Br(3)	14(1)	22(1)	25(1)	3(1)	3(1)	−4(1)
Br(4)	31(1)	12(1)	26(1)	7(1)	−10(1)	0(1)
Br(5)	16(1)	10(1)	18(1)	0(1)	1(1)	1(1)
Br(6)	10(1)	19(1)	24(1)	0(1)	0(1)	−1(1)
C(1)	12(2)	15(2)	9(2)	−2(2)	2(2)	2(2)
C(2)	16(2)	17(2)	8(2)	−1(2)	−1(2)	3(2)
C(3)	14(2)	17(2)	12(2)	−3(2)	1(2)	−1(2)
C(4)	14(2)	12(2)	17(2)	0(2)	1(2)	−4(2)
C(5)	10(2)	10(2)	18(2)	1(2)	2(2)	−2(2)
C(6)	19(2)	9(2)	13(2)	6(2)	−2(2)	4(2)
C(7)	13(2)	14(2)	11(2)	1(2)	−4(2)	0(2)
C(8)	12(2)	13(2)	12(2)	0(2)	0(2)	−1(2)
C(9)	12(2)	7(2)	15(2)	0(2)	−3(2)	4(1)
C(10)	7(2)	11(2)	23(2)	1(2)	0(2)	0(2)
C(11)	15(2)	11(2)	14(2)	−1(2)	3(2)	−2(2)
C(12)	16(2)	14(2)	13(2)	1(2)	−2(2)	3(2)

Table S5. Hydrogen coordinates (×10^4) and isotropic displacement parameters (Å²x 10 ^{3}) for (+)-α-HBCDD.

	x	y	z	U(eq)
H(1A)	7413	5506	8000	14
H(2A)	4320	5775	7702	16
H(3A)	3308	4217	7482	17
H(3B)	5257	3919	7097	17
H(4A)	6796	4045	8279	17
H(4B)	5101	3293	8343	17
H(5A)	4410	5234	8771	15
H(6A)	5096	4907	10,081	16
H(7A)	8344	4997	9110	15
H(7B)	8365	5258	9999	15
H(8A)	6279	6332	8879	15
H(8B)	6334	6598	9767	15
H(9A)	9619	6910	9662	14
H(10A)	10115	6014	8471	16
H(11A)	10377	6864	7334	16
H(11B)	9458	7807	7703	16
H(12A)	6512	7119	7703	17
H(12B)	7367	6974	6869	17

Table S6. Torsion angles [°] for (+)-α-HBCDD.

C(12)-C(1)-C(2)-C(3)	−171.8(3)
Br(1)-C(1)-C(2)-C(3)	59.1(4)
C(12)-C(1)-C(2)-Br(2)	62.3(4)
Br(1)-C(1)-C(2)-Br(2)	−66.8(3)
C(1)-C(2)-C(3)-C(4)	50.0(4)
Br(2)-C(2)-C(3)-C(4)	176.9(2)
C(2)-C(3)-C(4)-C(5)	58.6(4)
C(3)-C(4)-C(5)-C(6)	−151.8(3)
C(3)-C(4)-C(5)-Br(3)	82.3(3)
C(4)-C(5)-C(6)-C(7)	70.0(4)
Br(3)-C(5)-C(6)-C(7)	−164.8(2)
C(4)-C(5)-C(6)-Br(4)	−51.7(4)
Br(3)-C(5)-C(6)-Br(4)	73.4(3)
C(5)-C(6)-C(7)-C(8)	61.5(4)
Br(4)-C(6)-C(7)-C(8)	−175.8(2)
C(6)-C(7)-C(8)-C(9)	−179.1(3)
C(7)-C(8)-C(9)-C(10)	66.2(4)
C(7)-C(8)-C(9)-Br(5)	−169.9(2)
C(8)-C(9)-C(10)-C(11)	81.3(4)
Br(5)-C(9)-C(10)-C(11)	−41.1(4)
C(8)-C(9)-C(10)-Br(6)	−157.6(2)
Br(5)-C(9)-C(10)-Br(6)	80.0(3)
C(9)-C(10)-C(11)-C(12)	−58.1(5)
Br(6)-C(10)-C(11)-C(12)	179.9(3)
C(2)-C(1)-C(12)-C(11)	167.4(3)
Br(1)-C(1)-C(12)-C(11)	−62.8(4)
C(10)-C(11)-C(12)-C(1)	−50.9(4)

Symmetry transformations used to generate equivalent atoms.

Table S7. Crystal data and structure refinement for (+)-γ-HBCDD.

**Empirical Formula**	**$C_{12}H_{18}Br_6$**
Formula weight	641.72
Temperature	147(2) K
Wavelength	0.71073 Å
Crystal system	Monoclinic
Space group	$P2_1$
Unit cell dimensions	a = 11.3429(17) Å α= 90°.<br>b = 10.7740(18) Å β= 95.856(5)°.<br>c = 14.386(2) Å γ= 90°.
Volume	1748.9(5) Å^3
Z	4
Density (calculated)	2.437 Mg/m^3
Absorption coefficient	13.757 mm^{-1}
F(000)	1200
Crystal size	0.150 x 0.100 x 0.040 mm^3
Theta range for data collection	1.423 to 27.531°.
Index ranges	−14 ≤ h ≤ 13, −13 ≤ k ≤ 13, −18 ≤ l ≤ 18
Reflections collected	25,665
Independent reflections	8035 [R(int) = 0.0480]

Completeness to theta = 25.242°	100.0%
Absorption correction	Semi-empirical from equivalents
Max. and min. transmission	0.7456 and 0.4774
Refinement method	Full-matrix least-squares on F^2
Data/restraints/parameters	8035/1/325
Goodness-of-fit on F^2	0.914
Final R indices [I>2sigma(I)]	R1 = 0.0318, wR2 = 0.0382
R indices (all data)	R1 = 0.0509, wR2 = 0.0413
Absolute structure parameter	−0.009(9)
Extinction coefficient	n/a
Largest diff. peak and hole	0.623 and −0.539 e.Å^{-3}
Flack paramter	−0.009(9)

Table S8. Atomic coordinates (×10^4) and equivalent isotropic displacement parameters (Å^{2}x 10^3) for (+)-γ-HBCDD. U(eq) is defined as one third of the trace of the orthogonalized U^{ij} tensor.

	x	y	z	U(eq)
Br(1A)	4633(1)	4725(1)	4392(1)	25(1)
Br(2A)	6559(1)	3123(1)	5974(1)	20(1)
Br(3A)	2889(1)	3167(1)	8786(1)	28(1)
Br(4A)	5753(1)	1594(1)	9404(1)	25(1)
Br(5A)	7676(1)	7721(1)	8746(1)	24(1)
Br(6A)	8932(1)	6273(1)	6828(1)	28(1)
C(1A)	5014(6)	5233(6)	5704(4)	16(2)
C(2A)	5231(5)	4131(6)	6345(4)	14(2)
C(3A)	4194(5)	3269(6)	6503(4)	17(2)
C(4A)	4468(5)	2569(6)	7417(4)	20(2)
C(5A)	4409(5)	3403(6)	8274(4)	18(2)
C(6A)	5415(5)	3327(6)	9050(4)	17(2)
C(7A)	6552(5)	3943(6)	8789(4)	17(2)
C(8A)	6483(5)	5372(6)	8795(4)	15(2)
C(9A)	7520(5)	5978(6)	8359(4)	16(2)
C(10A)	7412(5)	5867(6)	7297(4)	14(2)
C(11A)	6428(5)	6620(6)	6761(4)	19(2)
C(12A)	6029(5)	6161(6)	5760(4)	18(2)
Br(1B)	−1233(1)	33(1)	4740(1)	27(1)
Br(2B)	1349(1)	1290(1)	6000(1)	24(1)
Br(3B)	2338(1)	−3994(1)	7575(1)	29(1)
Br(4B)	4401(1)	−1489(1)	8258(1)	23(1)
Br(7B)	−776(1)	775(1)	9966(1)	28(1)
Br(8B)	−394(1)	3181(1)	8239(1)	28(1)
C(1B)	−787(5)	−143(6)	6087(4)	18(2)
C(2B)	542(5)	−249(6)	6310(4)	18(2)
C(3B)	1157(5)	−1366(6)	5925(4)	16(2)
C(4B)	2300(5)	−1672(6)	6551(4)	18(2)
C(5B)	2056(6)	−2195(6)	7502(4)	18(2)
C(6B)	2692(5)	−1602(6)	8377(4)	17(2)
C(7B)	2221(5)	−313(6)	8583(4)	17(2)
C(8B)	1021(5)	−389(6)	9011(4)	18(2)
C(9B)	447(5)	903(6)	9086(4)	17(2)
C(10B)	−77(5)	1397(6)	8148(4)	16(2)
C(11B)	−1173(5)	738(6)	7677(4)	20(2)

C(12B)	−1382(5)	878(6)	6605(4)	19(2)

Table S9. Bond lengths [Å] and angles [°] for (+)-γ-HBCDD.

Br(1A)-C(1A)	1.970(6)
Br(2A)-C(2A)	1.975(6)
Br(3A)-C(5A)	1.960(5)
Br(4A)-C(6A)	1.963(6)
Br(5A)-C(9A)	1.961(6)
Br(6A)-C(10A)	1.963(5)
C(1A)-C(2A)	1.508(8)
C(1A)-C(12A)	1.521(8)
C(1A)-H(1AA)	1.0000
C(2A)-C(3A)	1.533(8)
C(2A)-H(2AA)	1.0000
C(3A)-C(4A)	1.520(8)
C(3A)-H(3AA)	0.9900
C(3A)-H(3AB)	0.9900
C(4A)-C(5A)	1.532(8)
C(4A)-H(4AA)	0.9900
C(4A)-H(4AB)	0.9900
C(5A)-C(6A)	1.515(8)
C(5A)-H(5AA)	1.0000
C(6A)-C(7A)	1.531(8)
C(6A)-H(6AA)	1.0000
C(7A)-C(8A)	1.541(8)
C(7A)-H(7AA)	0.9900
C(7A)-H(7AB)	0.9900
C(8A)-C(9A)	1.534(8)
C(8A)-H(8AA)	0.9900
C(8A)-H(8AB)	0.9900
C(9A)-C(10A)	1.526(8)
C(9A)-H(9AA)	1.0000
C(10A)-C(11A)	1.524(8)
C(10A)-H(10A)	1.0000
C(11A)-C(12A)	1.547(8)
C(11A)-H(11A)	0.9900
C(11A)-H(11B)	0.9900
C(12A)-H(12A)	0.9900
C(12A)-H(12B)	0.9900
Br(1B)-C(1B)	1.961(6)
Br(2B)-C(2B)	1.967(6)
Br(3B)-C(5B)	1.966(6)
Br(4B)-C(6B)	1.966(6)
Br(7B)-C(9B)	1.976(6)
Br(8B)-C(10B)	1.962(6)
C(1B)-C(2B)	1.514(8)
C(1B)-C(12B)	1.524(8)
C(1B)-H(1BA)	1.0000
C(2B)-C(3B)	1.523(8)
C(2B)-H(2BA)	1.0000
C(3B)-C(4B)	1.538(8)
C(3B)-H(3BA)	0.9900

C(3B)-H(3BB)	0.9900
C(4B)-C(5B)	1.530(8)
C(4B)-H(4BA)	0.9900
C(4B)-H(4BB)	0.9900
C(5B)-C(6B)	1.526(8)
C(5B)-H(5BA)	1.0000
C(6B)-C(7B)	1.528(8)
C(6B)-H(6BA)	1.0000
C(7B)-C(8B)	1.553(7)
C(7B)-H(7BA)	0.9900
C(7B)-H(7BB)	0.9900
C(8B)-C(9B)	1.546(9)
C(8B)-H(8BA)	0.9900
C(8B)-H(8BB)	0.9900
C(9B)-C(10B)	1.514(8)
C(9B)-H(9BA)	1.0000
C(10B)-C(11B)	1.529(8)
C(10B)-H(10B)	1.0000
C(11B)-C(12B)	1.544(8)
C(11B)-H(11C)	0.9900
C(11B)-H(11D)	0.9900
C(12B)-H(12C)	0.9900
C(12B)-H(12D)	0.9900
C(2A)-C(1A)-C(12A)	114.2(5)
C(2A)-C(1A)-Br(1A)	111.9(4)
C(12A)-C(1A)-Br(1A)	108.9(4)
C(2A)-C(1A)-H(1AA)	107.2
C(12A)-C(1A)-H(1AA)	107.2
Br(1A)-C(1A)-H(1AA)	107.2
C(1A)-C(2A)-C(3A)	119.2(5)
C(1A)-C(2A)-Br(2A)	110.4(4)
C(3A)-C(2A)-Br(2A)	109.0(4)
C(1A)-C(2A)-H(2AA)	105.8
C(3A)-C(2A)-H(2AA)	105.8
Br(2A)-C(2A)-H(2AA)	105.8
C(4A)-C(3A)-C(2A)	109.7(5)
C(4A)-C(3A)-H(3AA)	109.7
C(2A)-C(3A)-H(3AA)	109.7
C(4A)-C(3A)-H(3AB)	109.7
C(2A)-C(3A)-H(3AB)	109.7
H(3AA)-C(3A)-H(3AB)	108.2
C(3A)-C(4A)-C(5A)	112.5(6)
C(3A)-C(4A)-H(4AA)	109.1
C(5A)-C(4A)-H(4AA)	109.1
C(3A)-C(4A)-H(4AB)	109.1
C(5A)-C(4A)-H(4AB)	109.1
H(4AA)-C(4A)-H(4AB)	107.8
C(6A)-C(5A)-C(4A)	118.0(5)
C(6A)-C(5A)-Br(3A)	109.9(4)
C(4A)-C(5A)-Br(3A)	109.7(4)
C(6A)-C(5A)-H(5AA)	106.1
C(4A)-C(5A)-H(5AA)	106.1

Br(3A)-C(5A)-H(5AA)	106.1
C(5A)-C(6A)-C(7A)	112.7(5)
C(5A)-C(6A)-Br(4A)	110.8(4)
C(7A)-C(6A)-Br(4A)	109.2(4)
C(5A)-C(6A)-H(6AA)	108.0
C(7A)-C(6A)-H(6AA)	108.0
Br(4A)-C(6A)-H(6AA)	108.0
C(6A)-C(7A)-C(8A)	112.7(5)
C(6A)-C(7A)-H(7AA)	109.0
C(8A)-C(7A)-H(7AA)	109.0
C(6A)-C(7A)-H(7AB)	109.0
C(8A)-C(7A)-H(7AB)	109.0
H(7AA)-C(7A)-H(7AB)	107.8
C(9A)-C(8A)-C(7A)	112.4(5)
C(9A)-C(8A)-H(8AA)	109.1
C(7A)-C(8A)-H(8AA)	109.1
C(9A)-C(8A)-H(8AB)	109.1
C(7A)-C(8A)-H(8AB)	109.1
H(8AA)-C(8A)-H(8AB)	107.9
C(10A)-C(9A)-C(8A)	112.9(5)
C(10A)-C(9A)-Br(5A)	110.8(4)
C(8A)-C(9A)-Br(5A)	110.0(4)
C(10A)-C(9A)-H(9AA)	107.6
C(8A)-C(9A)-H(9AA)	107.6
Br(5A)-C(9A)-H(9AA)	107.6
C(11A)-C(10A)-C(9A)	116.4(5)
C(11A)-C(10A)-Br(6A)	109.3(4)
C(9A)-C(10A)-Br(6A)	110.1(4)
C(11A)-C(10A)-H(10A)	106.9
C(9A)-C(10A)-H(10A)	106.9
Br(6A)-C(10A)-H(10A)	106.9
C(10A)-C(11A)-C(12A)	115.5(5)
C(10A)-C(11A)-H(11A)	108.4
C(12A)-C(11A)-H(11A)	108.4
C(10A)-C(11A)-H(11B)	108.4
C(12A)-C(11A)-H(11B)	108.4
H(11A)-C(11A)-H(11B)	107.5
C(1A)-C(12A)-C(11A)	114.0(5)
C(1A)-C(12A)-H(12A)	108.7
C(11A)-C(12A)-H(12A)	108.7
C(1A)-C(12A)-H(12B)	108.7
C(11A)-C(12A)-H(12B)	108.7
H(12A)-C(12A)-H(12B)	107.6
C(2B)-C(1B)-C(12B)	115.6(6)
C(2B)-C(1B)-Br(1B)	111.5(4)
C(12B)-C(1B)-Br(1B)	109.3(4)
C(2B)-C(1B)-H(1BA)	106.6
C(12B)-C(1B)-H(1BA)	106.6
Br(1B)-C(1B)-H(1BA)	106.6
C(1B)-C(2B)-C(3B)	117.8(6)
C(1B)-C(2B)-Br(2B)	111.4(5)
C(3B)-C(2B)-Br(2B)	110.0(4)

C(1B)-C(2B)-H(2BA)	105.6
C(3B)-C(2B)-H(2BA)	105.6
Br(2B)-C(2B)-H(2BA)	105.6
C(2B)-C(3B)-C(4B)	110.3(5)
C(2B)-C(3B)-H(3BA)	109.6
C(4B)-C(3B)-H(3BA)	109.6
C(2B)-C(3B)-H(3BB)	109.6
C(4B)-C(3B)-H(3BB)	109.6
H(3BA)-C(3B)-H(3BB)	108.1
C(5B)-C(4B)-C(3B)	112.6(5)
C(5B)-C(4B)-H(4BA)	109.1
C(3B)-C(4B)-H(4BA)	109.1
C(5B)-C(4B)-H(4BB)	109.1
C(3B)-C(4B)-H(4BB)	109.1
H(4BA)-C(4B)-H(4BB)	107.8
C(6B)-C(5B)-C(4B)	118.0(5)
C(6B)-C(5B)-Br(3B)	108.0(4)
C(4B)-C(5B)-Br(3B)	111.6(4)
C(6B)-C(5B)-H(5BA)	106.2
C(4B)-C(5B)-H(5BA)	106.2
Br(3B)-C(5B)-H(5BA)	106.2
C(5B)-C(6B)-C(7B)	113.3(5)
C(5B)-C(6B)-Br(4B)	110.0(4)
C(7B)-C(6B)-Br(4B)	109.1(4)
C(5B)-C(6B)-H(6BA)	108.1
C(7B)-C(6B)-H(6BA)	108.1
Br(4B)-C(6B)-H(6BA)	108.1
C(6B)-C(7B)-C(8B)	111.5(5)
C(6B)-C(7B)-H(7BA)	109.3
C(8B)-C(7B)-H(7BA)	109.3
C(6B)-C(7B)-H(7BB)	109.3
C(8B)-C(7B)-H(7BB)	109.3
H(7BA)-C(7B)-H(7BB)	108.0
C(9B)-C(8B)-C(7B)	111.9(5)
C(9B)-C(8B)-H(8BA)	109.2
C(7B)-C(8B)-H(8BA)	109.2
C(9B)-C(8B)-H(8BB)	109.2
C(7B)-C(8B)-H(8BB)	109.2
H(8BA)-C(8B)-H(8BB)	107.9
C(10B)-C(9B)-C(8B)	112.6(5)
C(10B)-C(9B)-Br(7B)	111.0(4)
C(8B)-C(9B)-Br(7B)	108.0(4)
C(10B)-C(9B)-H(9BA)	108.4
C(8B)-C(9B)-H(9BA)	108.4
Br(7B)-C(9B)-H(9BA)	108.4
C(9B)-C(10B)-C(11B)	117.3(5)
C(9B)-C(10B)-Br(8B)	110.1(4)
C(11B)-C(10B)-Br(8B)	109.8(4)
C(9B)-C(10B)-H(10B)	106.3
C(11B)-C(10B)-H(10B)	106.3
Br(8B)-C(10B)-H(10B)	106.3
C(10B)-C(11B)-C(12B)	115.6(5)

C(10B)-C(11B)-H(11C)	108.4
C(12B)-C(11B)-H(11C)	108.4
C(10B)-C(11B)-H(11D)	108.4
C(12B)-C(11B)-H(11D)	108.4
H(11C)-C(11B)-H(11D)	107.4
C(1B)-C(12B)-C(11B)	112.8(5)
C(1B)-C(12B)-H(12C)	109.0
C(11B)-C(12B)-H(12C)	109.0
C(1B)-C(12B)-H(12D)	109.0
C(11B)-C(12B)-H(12D)	109.0
H(12C)-C(12B)-H(12D)	107.8

Symmetry transformations used to generate equivalent atoms.

Table S10. Anisotropic displacement parameters ($Å^2$x 10^3) for (+)-γ-HBCDD. The anisotropic displacement factor exponent takes the form: $-2\pi^2[h^2 a^{*2}U^{11} + ... + 2 h k a^* b^* U^{12}]$.

	U^{11}	U^{22}	U^{33}	U^{23}	U^{13}	U^{12}
Br(1A)	34(1)	24(1)	15(1)	0(1)	−4(1)	4(1)
Br(2A)	19(1)	18(1)	25(1)	0(1)	5(1)	5(1)
Br(3A)	19(1)	29(1)	38(1)	4(1)	13(1)	−2(1)
Br(4A)	27(1)	19(1)	28(1)	9(1)	1(1)	−1(1)
Br(5A)	31(1)	19(1)	24(1)	−5(1)	4(1)	−7(1)
Br(6A)	24(1)	31(1)	32(1)	4(1)	14(1)	−5(1)
C(1A)	24(4)	13(4)	11(3)	−3(3)	0(3)	0(3)
C(2A)	17(4)	17(4)	10(3)	−5(3)	4(3)	6(3)
C(3A)	12(3)	21(4)	17(4)	−4(3)	−2(3)	0(3)
C(4A)	15(4)	19(4)	26(4)	2(3)	5(3)	−1(3)
C(5A)	13(3)	19(4)	21(4)	3(3)	7(3)	−5(3)
C(6A)	17(3)	14(4)	20(4)	3(3)	7(3)	2(3)
C(7A)	18(4)	18(4)	13(4)	4(3)	−1(3)	−1(3)
C(8A)	16(3)	16(4)	14(4)	0(3)	1(3)	−5(3)
C(9A)	20(3)	11(4)	15(3)	−4(3)	0(3)	−3(3)
C(10A)	14(3)	12(3)	18(4)	1(3)	7(3)	−6(3)
C(11A)	24(4)	14(4)	20(4)	2(3)	6(3)	−1(3)
C(12A)	27(4)	12(4)	16(4)	3(3)	0(3)	2(3)
Br(1B)	24(1)	36(1)	20(1)	6(1)	−7(1)	−2(1)
Br(2B)	22(1)	26(1)	23(1)	4(1)	3(1)	−7(1)
Br(3B)	36(1)	17(1)	31(1)	−5(1)	−4(1)	8(1)
Br(4B)	14(1)	30(1)	26(1)	−6(1)	0(1)	6(1)
Br(7B)	26(1)	35(1)	27(1)	0(1)	14(1)	6(1)
Br(8B)	22(1)	14(1)	48(1)	−3(1)	4(1)	5(1)
C(1B)	18(4)	18(4)	16(4)	4(3)	−1(3)	−5(3)
C(2B)	19(4)	21(4)	14(3)	6(3)	0(3)	−3(3)
C(3B)	23(4)	14(4)	12(4)	−3(3)	5(3)	0(3)
C(4B)	18(4)	25(4)	12(3)	−2(3)	2(3)	9(3)
C(5B)	17(4)	16(4)	19(4)	0(3)	−4(3)	9(3)
C(6B)	11(3)	21(4)	19(4)	−2(3)	4(3)	2(3)
C(7B)	13(3)	16(4)	21(4)	−2(3)	−1(3)	−1(3)
C(8B)	24(4)	17(4)	16(4)	2(3)	6(3)	1(3)
C(9B)	13(3)	17(4)	21(4)	−2(3)	5(3)	3(3)
C(10B)	13(3)	11(4)	24(4)	−3(3)	8(3)	7(3)
C(11B)	14(4)	17(4)	28(4)	−1(3)	3(3)	3(3)
C(12B)	13(3)	19(4)	22(4)	5(3)	−6(3)	−2(3)

Table S11. Hydrogen coordinates (×10[4]) and isotropic displacement parameters (Å²x 10 ³) for (+)-γ-HBCDD.

	x	y	z	U(eq)
H(1AA)	4305	5680	5897	19
H(2AA)	5510	4482	6973	17
H(3AA)	3462	3763	6525	20
H(3AB)	4064	2673	5979	20
H(4AA)	5270	2202	7436	24
H(4AB)	3894	1881	7443	24
H(5AA)	4406	4276	8037	21
H(6AA)	5161	3769	9608	20
H(7AA)	6710	3660	8159	20
H(7AB)	7225	3673	9237	20
H(8AA)	5729	5637	8445	18
H(8AB)	6482	5664	9448	18
H(9AA)	8261	5539	8610	19
H(10A)	7247	4974	7144	17
H(11A)	6700	7491	6724	23
H(11B)	5731	6618	7123	23
H(12A)	5783	6886	5364	22
H(12B)	6714	5771	5499	22
H(1BA)	−1133	−947	6277	21
H(2BA)	679	−327	7005	22
H(3BA)	1350	−1183	5283	19
H(3BB)	618	−2091	5896	19
H(4BA)	2766	−2285	6229	22
H(4BB)	2783	−909	6649	22
H(5BA)	1190	−2076	7546	22
H(6BA)	2579	−2152	8921	20
H(7BA)	2116	174	7997	20
H(7BB)	2808	123	9024	20
H(8BA)	472	−934	8616	22
H(8BB)	1150	−766	9640	22
H(9BA)	1071	1496	9352	20
H(10B)	553	1310	7715	19
H(11C)	−1879	1060	7951	24
H(11D)	−1107	−157	7829	24
H(12C)	−2245	864	6412	22
H(12D)	−1073	1693	6425	22

Table S12. Torsion angles [°] for (+)-γ-HBCDD.

C(12A)-C(1A)-C(2A)-C(3A)	167.8(5)
Br(1A)-C(1A)-C(2A)-C(3A)	−67.9(6)
C(12A)-C(1A)-C(2A)-Br(2A)	−64.9(6)
Br(1A)-C(1A)-C(2A)-Br(2A)	59.4(5)
C(1A)-C(2A)-C(3A)-C(4A)	−157.9(5)
Br(2A)-C(2A)-C(3A)-C(4A)	74.2(5)
C(2A)-C(3A)-C(4A)-C(5A)	72.0(6)
C(3A)-C(4A)-C(5A)-C(6A)	−133.0(6)
C(3A)-C(4A)-C(5A)-Br(3A)	100.0(5)
C(4A)-C(5A)-C(6A)-C(7A)	73.5(7)
Br(3A)-C(5A)-C(6A)-C(7A)	−159.6(4)

C(4A)-C(5A)-C(6A)-Br(4A)	−49.1(6)
Br(3A)-C(5A)-C(6A)-Br(4A)	77.8(4)
C(5A)-C(6A)-C(7A)-C(8A)	74.0(7)
Br(4A)-C(6A)-C(7A)-C(8A)	−162.4(4)
C(6A)-C(7A)-C(8A)-C(9A)	−169.2(5)
C(7A)-C(8A)-C(9A)-C(10A)	74.1(7)
C(7A)-C(8A)-C(9A)-Br(5A)	−161.5(4)
C(8A)-C(9A)-C(10A)-C(11A)	69.3(7)
Br(5A)-C(9A)-C(10A)-C(11A)	−54.7(6)
C(8A)-C(9A)-C(10A)-Br(6A)	−165.6(4)
Br(5A)-C(9A)-C(10A)-Br(6A)	70.4(5)
C(9A)-C(10A)-C(11A)-C(12A)	−158.6(5)
Br(6A)-C(10A)-C(11A)-C(12A)	75.9(6)
C(2A)-C(1A)-C(12A)-C(11A)	−53.2(7)
Br(1A)-C(1A)-C(12A)-C(11A)	−179.1(4)
C(10A)-C(11A)-C(12A)-C(1A)	90.9(6)
C(12B)-C(1B)-C(2B)-C(3B)	171.2(5)
Br(1B)-C(1B)-C(2B)-C(3B)	−63.1(7)
C(12B)-C(1B)-C(2B)-Br(2B)	−60.3(6)
Br(1B)-C(1B)-C(2B)-Br(2B)	65.4(5)
C(1B)-C(2B)-C(3B)-C(4B)	−153.3(5)
Br(2B)-C(2B)-C(3B)-C(4B)	77.6(5)
C(2B)-C(3B)-C(4B)-C(5B)	69.8(7)
C(3B)-C(4B)-C(5B)-C(6B)	−129.0(6)
C(3B)-C(4B)-C(5B)-Br(3B)	105.1(5)
C(4B)-C(5B)-C(6B)-C(7B)	72.2(7)
Br(3B)-C(5B)-C(6B)-C(7B)	−160.2(4)
C(4B)-C(5B)-C(6B)-Br(4B)	−50.2(6)
Br(3B)-C(5B)-C(6B)-Br(4B)	77.4(4)
C(5B)-C(6B)-C(7B)-C(8B)	76.4(7)
Br(4B)-C(6B)-C(7B)-C(8B)	−160.7(4)
C(6B)-C(7B)-C(8B)-C(9B)	−171.5(5)
C(7B)-C(8B)-C(9B)-C(10B)	74.2(7)
C(7B)-C(8B)-C(9B)-Br(7B)	−162.9(4)
C(8B)-C(9B)-C(10B)-C(11B)	68.8(7)
Br(7B)-C(9B)-C(10B)-C(11B)	−52.5(6)
C(8B)-C(9B)-C(10B)-Br(8B)	−164.8(4)
Br(7B)-C(9B)-C(10B)-Br(8B)	74.0(5)
C(9B)-C(10B)-C(11B)-C(12B)	−156.0(5)
Br(8B)-C(10B)-C(11B)-C(12B)	77.4(6)
C(2B)-C(1B)-C(12B)-C(11B)	−58.5(7)
Br(1B)-C(1B)-C(12B)-C(11B)	174.7(4)
C(10B)-C(11B)-C(12B)-C(1B)	90.2(7)

Symmetry transformations used to generate equivalent atoms.
